# Radar sensor based machine learning approach for precise vehicle position estimation

**DOI:** 10.1038/s41598-023-40961-5

**Published:** 2023-08-24

**Authors:** Muhammad Sohail, Abd Ullah Khan, Moid Sandhu, Ijaz Ali Shoukat, Mohsin Jafri, Hyundong Shin

**Affiliations:** 1 Riphah College of Computing, Riphah International University Faisalabad, Faisalabad, Pakistan; 2 Department of Computer Science, National University of Sciences and Technology Balochistan Campus, Quetta, Pakistan; 3 Australian e-Health Research Centre, Commonwealth Scientific & Industrial Research Organization (CSIRO), Brisbane, Australia; 4 Department of Electronics and Information Convergence Engineering, Kyung Hee University, Youngin-si, South Korea

**Keywords:** Computer science, Information technology

## Abstract

Estimating vehicles’ position precisely is essential in Vehicular Adhoc Networks (VANETs) for their safe, autonomous, and reliable operation. The conventional approaches used for vehicles’ position estimation, like Global Positioning System (GPS) and Global Navigation Satellite System (GNSS), pose significant data delays and data transmission errors, which render them ineffective in achieving precision in vehicles’ position estimation, especially under dynamic environments. Moreover, the existing radar-based approaches proposed for position estimation utilize the static values of range and azimuth, which make them inefficient in highly dynamic environments. In this paper, we propose a radar-based relative vehicle positioning estimation method. In the proposed method, the dynamic range and azimuth of a Frequency Modulated Continuous Wave radar is utilized to precisely estimate a vehicle’s position. In the position estimation process, the speed of the vehicle equipped with the radar sensor, called the reference vehicle, is considered such that a change in the vehicle’s speed changes the range and azimuth of the radar sensor. For relative position estimation, the distance and relative speed between the reference vehicle and a nearby vehicle are used. To this end, only those vehicles are considered that have a higher possibility of coming in contact with the reference vehicle. The data recorded by the radar sensor is subsequently utilized to calculate the precision and intersection Over Union (IOU) values. You Only Look Once (YOLO) version 4 is utilized to calculate precision and IOU values from the data captured using the radar sensor. The performance is evaluated under various real-time traffic scenarios in a MATLAB-based simulator. Results show that our proposed method achieves 80.0% precision in position estimation and obtains an IOU value up to 87.14%, thereby outperforming the state-of-the-art.

## Introduction

**Figure 1:**
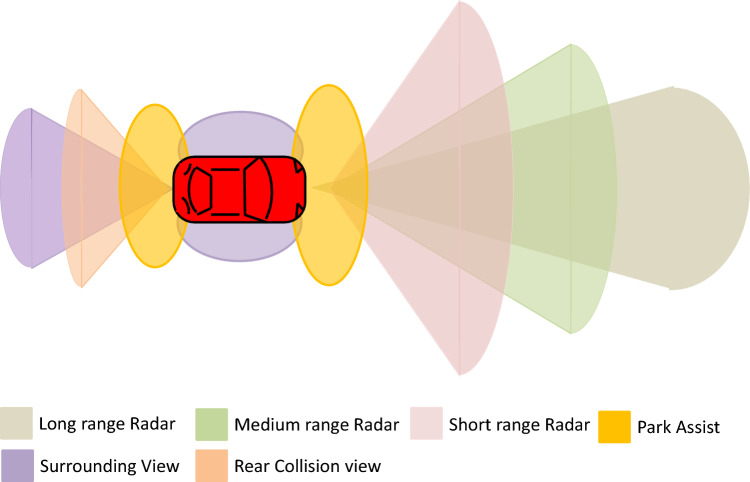
A reference vehicle equipped with multiple sensors.

Driverless vehicles are the future of transportation to make human life easy and reduce the number of road accidents due to human error^1^. These vehicles over the roads perform decision making without human intervention. Every automated vehicle takes decisions with respect to its environment, position, and direction of movement. However, these automated vehicles are still not intelligent enough to rely completely on their power of decision making which increases the risk of financial losses and deaths^2^. A vehicle, during its visit over the road, requires a wide range of parameters for its successful operation which includes position of vehicle, direction of motion, speed, the time of joining the network, and expected time to stay within that network^3^. All these parameters help the vehicle to take decisions during its operation on the road. Automated vehicles over the road communicate with other vehicles using two different approaches, i.e., Vehicle to Vehicle (V2V) and Vehicle to Infrastructure (V2I) communication^4^. In V2V communication, vehicles transmit their data directly from one vehicle to the other within the network. On the other hand, in V2I communication, a vehicle sends its data to Road Side Units (RSUs) or base station which is then sent to other vehicles in the network for safe travelling. Among all aforementioned core parameters of Vehicular Adhoc Networks (VANETs), the most important parameter for communication is location/position of vehicle. In Advanced Driving Assistance Systems (ADAS), different types of sensors i.e., infrared cameras, LiDAR, short range (≤ 50 m), medium range (≤ 100 m) and long range (≤ 250 m) radars are used to access the position of a vehicle in the surroundings of a reference vehicle^5^. ADAS have recently become the key research area to devise a perfect system that simulates human behavior. Figure 1 shows an ADAS system where reference vehicle is equipped with multiple sensors to get detailed information about the surrounding environment for collision avoidance^6^. A vehicle having sensors to detect its surroundings is named as reference vehicle. Every sensor has its own advantages and limitations based on its inherent characteristics and type of usage. Cameras give good semantic understandings of environment; however, they are not robust enough to perform well in extreme weather conditions i.e., fog, rain, and lightning^7^. LiDAR sensor is also ineffective in heavy rains and cloudy weather^8^. It generates large amount of data^9^ that needs more computational resources and complicated data/task management procedures. Frequency Modulated Continuous Wave (FMCW) radar is a favourable choice for automated vehicles^10–12^ due to its outstanding performance in harsh weather conditions, less cost and the ability to generate moderate amount of data which reduces the computational complexity.

In this paper, we propose a position estimation (position estimation is interchangeably used with position calculation) method based on dynamic range and azimuth value of radar sensor, that provides accurate information for vehicle position estimation. The inter communication of vehicles is improved based on dynamic range and azimuth value of radar sensor mounted on a reference vehicle. The value of azimuth and range of radar sensor is dependent on the speed of the reference vehicle. The slower speed of reference vehicle results in smaller range and higher azimuth value of radar however, its higher speed results in a larger range and a lower azimuth value of radar. The proposed strategy improves precision and Intersection Over Union (IOU) value in VANETs.

Rest of the paper is organised as follows: Literature review is presented in Section “Background”. Section “Motivation” discusses the problem motivation and also presents the major contribution of this research work. Section “Methodology” discusses the proposed research methodology in detail. Section “Range and Azimuth parameters” is all about implementation of proposed method. The results are discussed in Section “Results and discussions” based on the proposed methodology in comparison with the already available studies. Finally, in Section “Conclusion and future work”, we conclude the paper, discuss its limitations and present the future directions.

## Background

In the following section, we discuss the existing methodologies for vehicle position estimation that employ various types of sensing mechanisms including radar, camera, LiDAR, and signal fusion.

### Radar sensor based methods

A radar sensor-based solution for object detection and classification is presented in^13^. Radar system works in two phases i.e., object detection and object classification. In this algorithm, researchers merge both of these stages and convert them into one. Data set captured from radar sensor is used for precision detection using *You Only Look Once (YOLO)* algorithm. Simulation results show that the proposed method performs well i.e., it can compute up to 46.16% radar precision. To devise a better radar-based solution for vehicle detection in ADAS is discussed in^14^. In this model, a radar sensor is used to detect the vehicles over the road. Simulation is performed on nuScenes data set^15^ while the results show 65.41% precision value which is reliable in real-time environment for ADAS.

In the automotive industry, object detection has always been a challenge. Researchers implement different strategies to detect objects for safe driving but still, there is a lot to do. To overcome the said problem, a method is proposed in^16^ to detect the object in ADAS. In this method, a radar sensor is used to detect a single object, and a post-processing structure is implemented to cluster and detect multiple objects. The proposed method is tested in different driving scenarios. High perception of the vehicular environment is the backbone of the automated traffic industry. Different methodologies are already available for traffic perception but they only perform object classification of bounding box detections. To overcome these limitations radar sensor-based solution is presented to detect 2D objects using PointNets in^17^. Researchers claim that their method performs object classification with bounding box detections using a single radar. PointNets are adjusted according to the detections done by radar. The algorithm is tested using an automatically generated traffic scenario data set which is a combination of real-time traffic scenario maneuvers. Simulation results show that the proposed method can detect object with high accuracy.

To overcome the high processing time consumption associated with region proposal algorithms for vehicle position estimation, a solution is proposed in^18^. Radar Region Proposal Network (RRPN) is a radar-based real-time region proposal algorithm for object detection. RRPN estimates the object’s position by mapping radar data on the image received from the camera. Anchor boxes are drawn over the image. These anchor boxes are scaled, based on the distance of the object from the reference vehicle. The strategy is tested on the NuScenes data set^15^. Experimental results show that RRPN performs 100 × better than Region-based Convolution Neural Networks (RCNN).

### Camera sensor based methods

A solution using a monocular camera utilizing visible light for communication is proposed in^19^, to find the V2V position with better accuracy. Baseline issues associated with monocular cameras are reduced in the proposed method by propagating the known length of tail light as the baseline for the camera. Kalman Filter (KF) is used to improve positioning accuracy and remove errors from noisy data. A mobile application is used to verify the performance of the algorithm. Different scenarios are generated by varying the speed of vehicles or by varying the distance between vehicles. Experimental results show that the proposed method attains significant accuracy for the positioning of vehicles.

In ADAS, road safety is an important issue. To improve road safety, a monocular camera-based strategy by measuring the position and size of the vehicle is presented in^20^. Inverse perspective mapping is used to determine the distance in the image captured from the camera. In this algorithm, Inertial Measurement Unit (IMU) is used to cancel the roll motion and pitch of the camera while Convolutional Neural Network (CNN) is used to measure the position, direction of motion, and size of the vehicle. For simulation, KITTI data set^21^ is used, while the results show that the proposed method outperforms already available neural networks for position estimation of vehicles. This algorithm cannot differentiate between different types of traffic i.e., pedestrians, cyclists, and vehicles that is its limitation.

Another positioning algorithm is presented in^22^ using a CMOS camera and visible light for V2V positioning based on a modified version of KF. Authors use light beams emitting from LEDs to get the position of vehicles in the network. Two CMOS cameras are used to receive these LED light signals. On detection of at least one light beam in both cameras, the position of the vehicle is estimated. The received results contain some random values and errors due to the usage of CMOS technology used in cameras. Authors detect vehicles using a CMOS camera and find errors in received data then, they apply modified KF to remove the errors and obtain smooth results. Results show that system performs better than previous camera-based techniques for vehicle position accuracy.

Distance measurement between two vehicles in autonomous driving systems is explained in^23^ using camera and visible light communication. The proposed method uses two vision sensors for image captioning and only one LED to estimate the distance between these vehicles. Less expensive cameras are used that have merely enough capability to determine the coordinates in an image captured during driving.

### LiDAR sensor based methods

LiDAR-based solutions for precise vehicle position estimation has been widely used by the research community^24^. For example, a LiDAR sensor-based solution for object detection over the roads in VANETs is discussed in^25^. Researchers present an improved programming approach by using a LiDAR sensor in which two variants of YOLO; tiny-YOLO and complex-YOLO, are compared. The average precision shown by complex-YOLO is much better than tiny-YOLO which could be further improved by implementing better hardware. Another approach to measuring the accurate vehicle position is presented in^26^ using 3D LiDAR and RGB images. CNN is utilized to extract information from RGB images. This model takes the benefit of accurate depth information utilizing LiDAR data and semantic information from the camera. The proposed method is tested using KITTI^21^ real-time data set for the vehicular environment. Simulation results show that the proposed algorithm outperforms as compared to already available CNN-based position estimation algorithms.

Vehicle speed and position estimation is the point of significance for ADAS. LiDAR technologies play a central role in the detection of vehicle position and speed by scanning a 3D environment. Researchers in^27^ propose a solution for speed and position estimation of vehicles using roadside LiDAR. By utilizing the proposed framework, first vehicles are detected using an observed point cloud, and later centroid-based tracking flow is used to find the vehicle’s initial transformation. A tracker is utilized along with KF for tracking flow. Simulation 94% vehicles can be detected by using the proposed algorithm. The authors in^28^ surveyed several state-of-the-art localization protocols used for VANETs.

### Sensor fusion based methods

To improve autonomous driving experience, a radar sensor-based solution is explained in^29^. A multisensory model including LiDAR, radar, camera, and Global navigation satellite system (GNSS) is introduced which provides information about elevation measurement as well as point cloud data. A deep learning approach is implemented on data set collected from these sensors for detection of 3D objects. Real-world data is collected from Suzhou, China for experimentation. Simulation results show that proposed method is efficient to distinguish between different objects i.e., car, truck, and cyclist over the roads.

In-time and accurate object detection is necessary for autonomous driving systems. Utilization of single sensor comes across many challenges in vehicle detection and finding the accurate position due to continuously changing traffic scenarios over the road. In order to overcome the single sensor challenges, camera and LiDAR based solution is presented in^30^. Authors first convert the 2D LiDAR data into dense depth map that is feasible for both sensor synchronization. After that, YOLO algorithm is applied to detect dense depth map. Bounding box fusion is applied to merge the results from both sensors. Experiments are performed using KITTI data set^21^ while the results show that algorithm works better than the single sensor algorithms for vehicle detection.

A method for object detection based on radar sensor cloud points and camera images is proposed in^31^. A deep learning convolutional network is trained using labeled bounding boxes to detect cars. The results are compared with LiDAR data for vehicle detection while the performance is compared in terms of average precision that goes up to 61.0%. Simulation results show that the method works better than LiDAR data for vehicle detection on the road. The only limitation associated with this work is the availability of a large data set for performance evaluation.

Researchers utilize a combination of radar and vision-based data in^32^ to find the location of distant objects in ADAS. In far vehicle detection scenarios, two vehicles having the same speed moving far apart is a challenge in ADAS because it is really difficult to detect whether a single or two vehicles are moving in parallel. Many camera-based solutions for object detection are already available based on CNN, but the performance of these networks decreases for far and small objects. Researchers solve this problem by proposing an algorithm that detects and differentiates easily between small and far objects moving on the road. Simulations are performed using a data set generated from cameras of different focal lengths recording traffic scenes. Results show that the proposed method is efficient in the detection of far and smaller objects.

A radar and camera-based algorithm for object detection in ADAS is experimented with in^33^, to evaluate the performance of the proposed solution. Radar Object Detection Network (RODNet) is presented by researchers to effectively detect objects from frequency radar-based data. To detect objects from each snapshot of RAMaps, a 3D auto-encoder-based architecture is utilized. The algorithm is trained using a Camera, and LiDAR fusion strategy. The authors use a custom data set having videos and RAMaps while the results show that the proposed method has better object detection results in ADAS. Table 1 summarizes the recorded literature that has focused on position estimation methods.

**Table 1:** Comparative analysis of position estimation methods .

References	Precision and IOU	Limitations
Zheng et al.^29^	Precision: 33.30% IOU value is not considered	Precision value is too low which needs to be improved
Kim et al.^13^	Precision: 46.16% IOU: 74.34%	IOU is comparatively better but precision is very low which needs improvement
Muckenhuber et al.^14^	Precision: 65.41% IOU value is not considered	Precision value is quite low which needs to be improved by considering IOU factor
Kim and Kum^20^	Precision: 62.25% IOU value is not considered	IOU value is not considered, precision value is also low which needs improvement
Dazlee et al.^25^	Precision: 75.4% IOU value is not considered	Precision value is low and researchers also have not considered IOU
Barea et al.^26^	Precision: 79.77% IOU value is not considered	Precision value is comparatively better but IOU is not considered by researchers which needs attention
Guan et al.^30^	Precision: 57.9%	Precision is calculated by using LiDAR and camera sensor, IOU factor is not considered
Meyer and Kuschk^31^	Precision: 61% IOU value is not considered	Precision is quite low which needs improvement along with IOU value
Chadwick et al.^32^	Precision: 50.6% IOU value is not considered	Precision value is very low i.e., only 50.6% which needs improvement

## Motivation

Researchers in^14^ utilize static range and azimuth value of radar sensors to find the accurate vehicle position. The discussed methodology offers a low value of precision and IOU parameters. Researchers present a camera sensor-based method^19^ to improve vehicle position estimation. However, due to associated limitations in the real-world environment, especially in harsh weather, camera sensor-based methods do not offer promising results. A LiDAR sensor-based method to find precise vehicle position is presented in^25^. LiDAR sensor gets fail in the rain and extreme weather conditions. Furthermore, the LiDAR sensor generates a heavy amount of data that takes significant time in processing. Due to these limitations, LiDAR sensor-based methods are not feasible in vehicular environments.

To overcome limitations in single sensor-based methods, researchers propose a sensor fusion-based method to find the precise vehicle position in^29^. Data synchronization is a major problem with these combinations of sensors. Sensor fusion is a complex task and data generated from multiple sensors are difficult to manage on the resource-constrained devices. Due to the aforementioned limitations, we propose a radar sensor-based method utilizing the dynamic range and azimuth value of the radar sensor. This method uses a single radar sensor which is more effective in real-world traffic scenarios and detects objects even in harsh weather conditions. Simulation results show that the proposed method accurately estimates the vehicle position and offers significantly better performance in terms of precision and IOU.

**Table 2:** Comparative analysis of previous vehicle positioning methods based on radar sensor parameters .

Literature	Radar range (dynamic/static)	Radar Azimuth (dynamic/static)	Object detection (2D/3D)	Sensor used
Usage of radar sensor for vehicle detection^29^	Static	Static	3D	Radar
Sensor based technology for object detection^25^	Static	Static	3D	LiDAR
Performance evaluation for radar approaches for object detection^14^	Static	Static	3D	Radar
Vehicle detection using camera and LiDAR^30^	Static	Static	2D	Camera and LiDAR
Low level sensor fusion for object detection^13^	Static	Static	3D	Radar and Camera
Object detection using radar^33^	Static	Static	3D	Radar
Object detection and classification using radar^34^	Static	Static	2D	Radar
Proposed method	Dynamic	Dynamic	3D	Radar

In Table 2, a comparison between the previous approaches and the proposed algorithm is presented based on the sensor used, radar range, radar azimuth, and object detection (2D/3D). Some of the approaches in the table employ 2D object detection which means that these techniques can only detect the height and width of a vehicle and are unable to consider the length of the vehicle. On the other hand, in 3D detection, the previous approaches not only detect the vehicle but also are efficient in measuring the height, width, and length of the vehicle.

### Major contribution of paper

In this paper, we propose a technique to improve the precision and IOU value for vehicle position estimation based on the dynamic values of range and azimuth for radar sensors. Our main contribution is given as:

1. We devise a computational framework for an improved vehicle detection algorithm using the dynamic value of range and azimuth value of the radar sensor. The range and azimuth value of the radar sensor merely depends upon the speed of the reference vehicle. If the speed of the reference vehicle is low, the range of the radar sensor decreases while the azimuth value increases and vice versa.
2. We design different real-time traffic scenarios utilizing *Driving scenario generator app* in which a reference vehicle is equipped with a radar sensor having static range and azimuth value. In these scenarios, the majority of the accidents occur due to the static range and azimuth value of radar sensor^29^.
3. Real-time data is generated against these simulation scenarios for radar sensors, which is used for the calculation of precision and IOU value using the YOLO algorithm^35^. The results are compared with the competitive methods utilizing static range and azimuth value of radar sensor.

## Methodology

**Figure 2:**
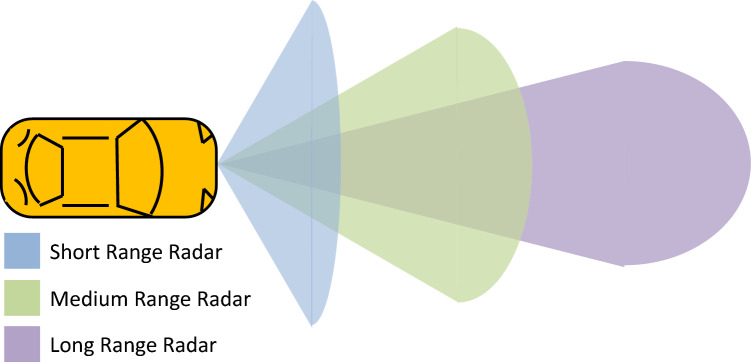
A reference vehicle using multiple radar sensors for vehicle detection.

**Figure 3:**
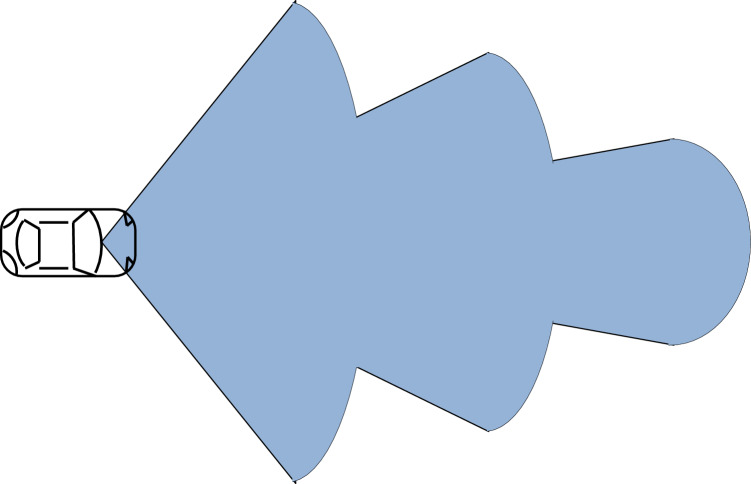
A reference vehicle equipped with a single radar sensor using dynamic range and azimuth value.

The proposed work computes the precise vehicle position using a single radar sensor mounted in front of the vehicle rather than using multiple radar sensors. Data synchronization from multiple radar sensors having fixed/static range and azimuth value is a major challenge in accurate vehicle position estimation^36^. To solve this issue, we employ a single radar sensor and use dynamic range and azimuth values that perform better in real-time traffic scenarios in terms of vehicle position estimation. The proposed method using a single radar offers better precision and IOU values. A vehicle using multiple radar sensors is shown in Fig. 2, whereas a vehicle utilizing a single radar sensor based on dynamic range and azimuth value is shown in Fig. 3.

**Figure 4:**
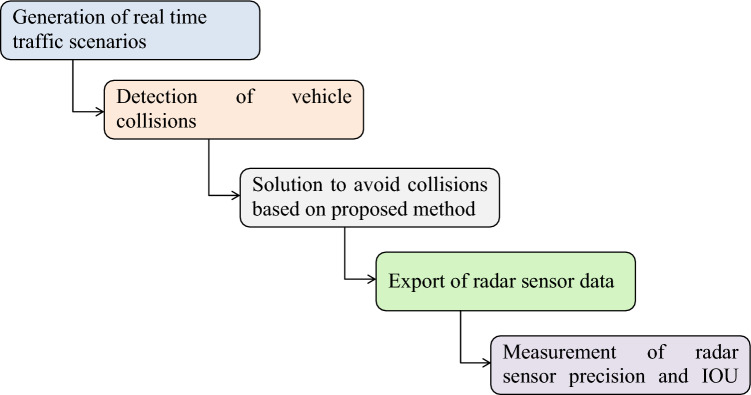
Framework of the proposed method.

The computational framework of our proposed method is shown in Fig. 4. In the first step, real-time traffic scenarios are generated using *Driving scenario generator app*, and vehicle collisions in these traffic scenarios are detected using the radar sensor. In the next step, the solutions for these failure scenarios are presented based on the proposed method i.e., utilizing dynamic range and azimuth value of radar. Radar sensor data is generated against these real-time traffic scenarios. In the next step, this radar sensor data is exported and utilized for precision and mean IOU value calculation of the radar sensor using the YOLO algorithm.

### Vehicle detection algorithm

**Figure 5:**
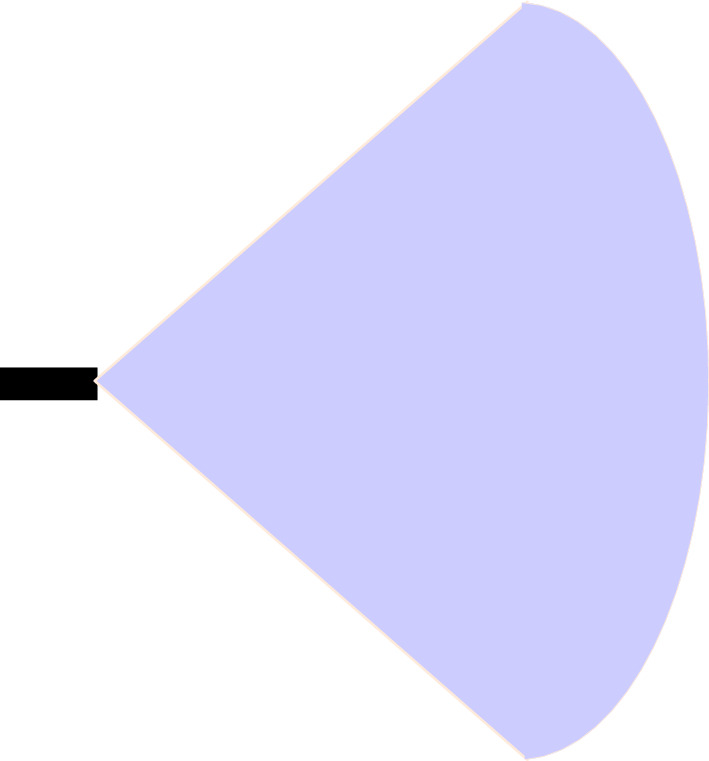
Speed of reference vehicle is less than 40 Km/h; range = 30 m, azimuth = 120°.

**Figure 6:**
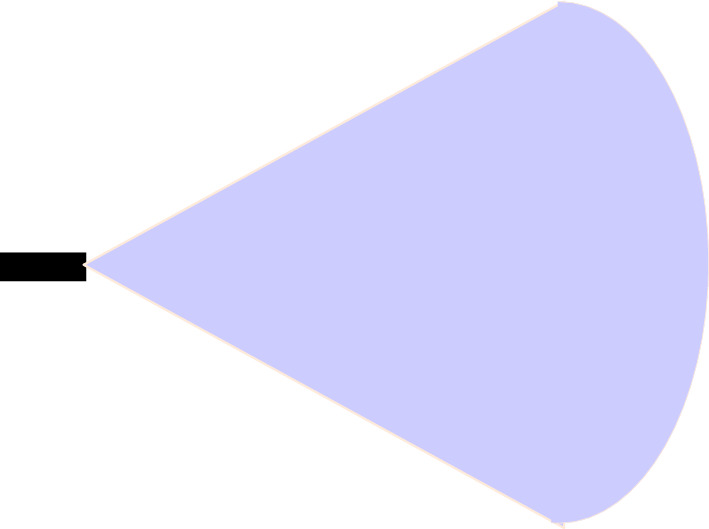
Speed of reference vehicle is less than 40 to 120 Km/h; range = 50 m, azimuth = 80°.

**Figure 7:**
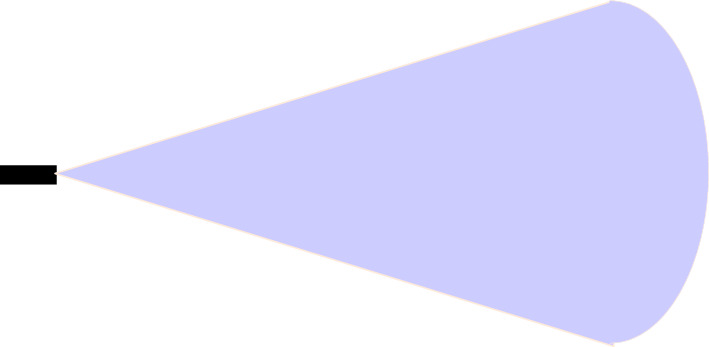
Speed of reference vehicle is 121 Km/h or more; range = 100 m, azimuth = 50°.

This algorithm is based on the dynamic range and azimuth value of radar sensors to detect vehicles over the road for collision avoidance in vehicular networks. Dynamic shifting of radar sensor range and azimuth value can be summarised below as shown in Figs. 5, 6, and 7:

- If the speed of the reference vehicle is S1; the range of the radar will be R1 and the azimuth value A1.
- If the speed of the reference vehicle is S2; the range of radar would be R2 and azimuth value A2.
- If the speed of the reference vehicle is S3; the range of radar would be R3 and azimuth value A3.

**Figure 8:**
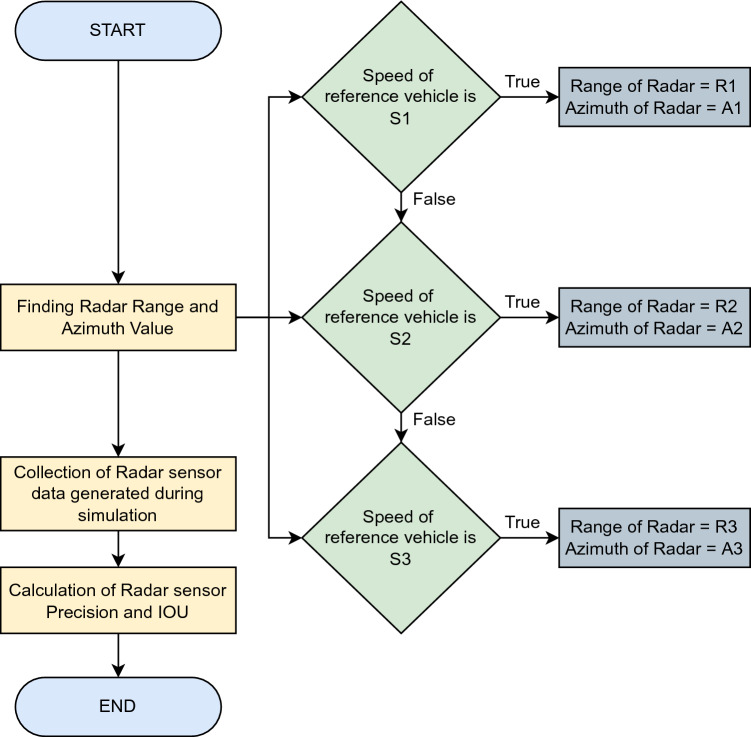
Design of the proposed method in a flowchart.

We present the proposed vehicle position estimation method in Fig. 8 and Algorithm 1. It is clear from the figure that the range and azimuth value of the radar sensor merely depends upon the speed of the reference vehicle. A real-time data set extracted from traffic scenarios is then fed to the YOLO algorithm for the calculation of average precision and mean IOU. The details of the parameters used in the proposed algorithm are as given in Table 3.

**Table 3:** Parameters.

Parameter	Speed of reference vehicle	Range of radar sensor	Azimuth value of radar sensor
S1	Speed is up to 40 Km/h	R1 = 30 m	A1 = 120°
S2	Speed is 41 Km/h to 120 Km/h	R2 = 50 m	A2 = 80°
S3	Speed is 121 Km/h or more	R3 = 100 m	A3 = 50°

Mathematically, the relationship between the range and azimuth value of a radar sensor could be written as:

1 $$\begin{aligned}{} & {} range \propto speed\; of\; reference\; vehicle \end{aligned}$$

2 $$\begin{aligned}{} & {} azimuth \propto \frac{1}{ speed\; of\; reference\; vehicle\;} \end{aligned}$$

From Eqs. (1) and (2), it is clear that range and azimuth value of radar sensor are dependant on the speed of the reference vehicle. If reference vehicle is moving at high speed range of radar sensor would be higher while azimuth value would be lower, and vice versa.
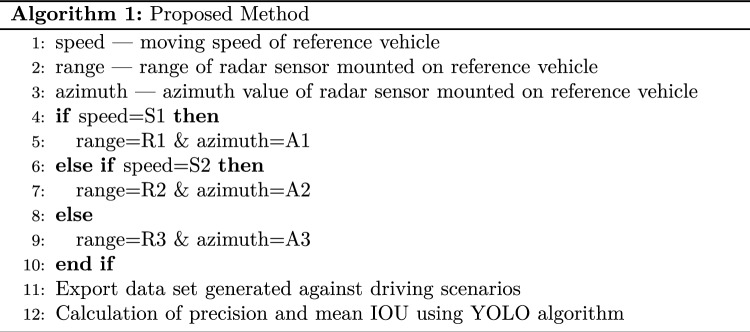


Radar sensor data is exported and utilized for precision and mean IOU value calculation of radar sensors using the YOLO algorithm.

### Brief description of YOLO

**Figure 9:**
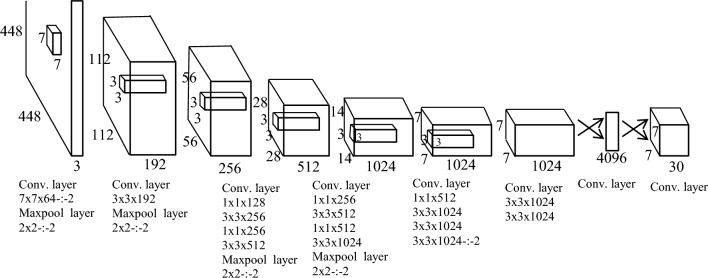
Architecture of YOLO algorithm.

YOLO is a CNN-based method for object detection and classification in real-time traffic scenarios^35^. YOLO algorithm needs single forward propagation for object detection which means prediction is done in a single go through the algorithm. The YOLO algorithm is based on the regression technique. Instead of selecting the intersection part of an image, it predicts the class as well as the bounding box for the whole image in a single iteration^37^. The detailed architecture of the YOLO algorithm is presented in Fig. 9^38^.

In YOLO, an image is divided into S × S grid cells^34^. Every grid cell is the combination of confidence score and B bounding boxes. This combination determines the probability of an object’s existence in that specific cell. The conditional probability parameter is also associated with every grid cell which is responsible to determine whether the object of every class exists or not. In YOLO, the confidence score for a specific class is obtained by the multiplication of conditional probability and confidence score. Finally, object detection results are computed by comparing class-specific confidence scores^34^.

YOLO utilizes the following three techniques for object detection^39–41^:

**Figure 10:**
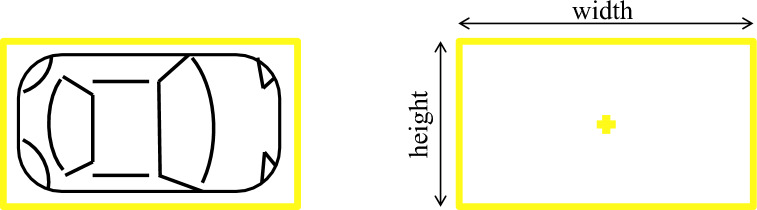
Bounding box regression example: yellow box representing a bounding box around an object.

**Figure 11:**
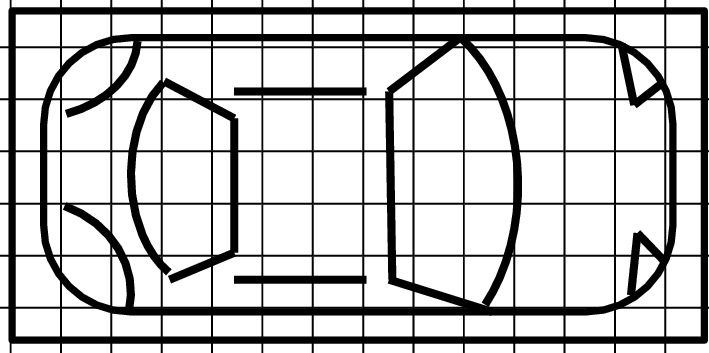
Residual blocks example: image is divided into grids.

**Figure 12:**
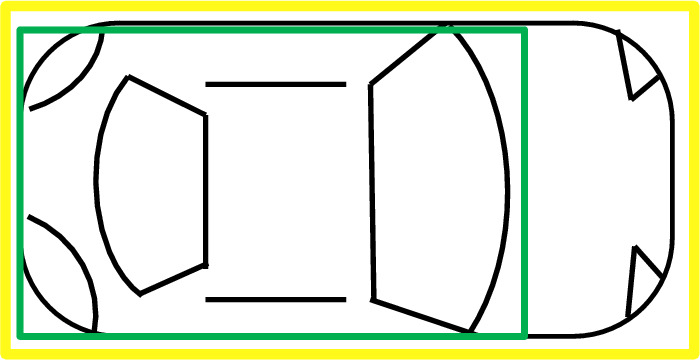
Intersection over union example: diagram showing predicted and real bounding box using IOU technique.

- *Bounding Box Regression*
  In an image bounding box could be defined as the outline around the detected object. Every bounding box around the object may have some attributes i.e., height, width, center, and class of the object. An example of the bounding box technique is shown in Fig. 10.
- *Residual Blocks*
  In residual blocks, an image is divided into grids and the dimensions of this grid can be written as S × S. An example of a grid around an object is shown in Fig. 11.
- *Intersection Over Union (IOU)*
  IOU could be defined as the process of object detection which explains how boxes overlap. YOLO is capable of making a bounding box around the detected object perfectly. Every bounding box is assigned a confidence score. If the predicted bounding box is the same as the real bounding box then the IOU value will be ‘1’. Bounding boxes that are not the same as real bounding boxes are eliminated in this mechanism. An example of the IOU mechanism is shown in Fig. 12 where the yellow box is the real bounding box while green is the predicted bounding box.

The loss function is the most important parameter of the YOLO algorithm which is optimized during training. The loss function is totally dependent on sum-squared error which can be written as^37^:

$$\begin{aligned}{} & {} \lambda _{coord} \sum \limits _{i=0}^{ \begin{array}{c} S^2 \end{array}} \sum \limits _{j=0}^{ \begin{array}{c} B \end{array}} \mathbbm {1}_{ij}^{obj} [(x_i - \hat{x})^2 + (y_i + \hat{y}_i)^2] \\{} & {} \quad + \lambda _{coord} \sum \limits _{i=0}^{ \begin{array}{c} S^2 \end{array}} \sum \limits _{j=0}^{\begin{array}{c} B \end{array}} \mathbbm {1}_{ij}^{obj}[(\sqrt{\omega }-\sqrt{\hat{\omega }})^2 +(\sqrt{\mathcalligra{h}_i} - \sqrt{\hat{\mathcalligra{h}_i}})^2] \\{} & {} \quad + \sum \limits _{i=0}^{ \begin{array}{c} S^2 \end{array}} \sum \limits _{j=0}^{ \begin{array}{c} B \end{array}} \mathbbm {1}_{ij}^{obj}[(C_i - \hat{C}_i)^2] \\{} & {} \quad + \lambda _{coord}\sum \limits _{i=0}^{ \begin{array}{c} S^2 \end{array}} \sum \limits _{j=0}^{ \begin{array}{c} B \end{array}} \mathbbm {1}_{ij}^{noobj}[(C_i - \hat{C}_i)^2] \\{} & {} \quad + \sum \limits _{i=0}^{ \begin{array}{c} S^2 \end{array}} \mathbbm {1}_{i}^{obj} \sum \limits _{c \in classes} (p_i(c)-\hat{p_i}(c))^2 \end{aligned}$$

Loss related to predicted bounding box position as well as ground truth bounding box position is computed in the first part of the equation $$\lambda _{coord} \sum \nolimits _{i=0}^{ \begin{array}{c} S^2 \end{array}} \sum \nolimits _{j=0}^{ \begin{array}{c} B \end{array}} \mathbbm {1}_{ij}^{obj} [(x_i - \hat{x})^2 + (y_i + \hat{y}_i)^2]$$ by using $(x_{center}, y_{center})$ coordinates^42^. ${\mathbbm {1}}_{i}^{obj}$ represents whether object exists in a specific cell of the grid made over detection *i* and ${\mathbbm {1}}_{ij}^{obj}$ represents that $j^{th}$ bounding box predictor is in that specific cell *i*. In the second part, loss function $$\lambda _{coord} \sum \nolimits _{i=0}^{ \begin{array}{c} S^2 \end{array}} \sum \nolimits _{j=0}^{ \begin{array}{c} B \end{array}} \mathbbm {1}_{ij}^{obj}[(\sqrt{\omega }-\sqrt{\hat{\omega }})^2 +(\sqrt{\mathcalligra{h}_i} - \sqrt{\hat{\mathcalligra{h}_i}})^2]$$ calculates the error in bounding box prediction. The confidence score is calculated whether an object is present or not within the bounding box. The loss function is responsible for object error in confidence. ${\mathbbm {1}}_{ij}^{obj}$ will have the value of ‘1’ if the object is present in the bounding box and otherwise, it will be ‘0’. The last part of the function $$\sum \nolimits _{i=0}^{ \begin{array}{c} S^2 \end{array}} \mathbbm {1}_{i}^{obj} \sum \nolimits _{c \in classes} (p_i(c)-\hat{p_i}(c))^2$$ is responsible for the class probability loss. Whenever there is no object, YOLO does not care about the classification error^42^. During training, this loss function is optimized. The loss function is only responsible for classification errors when an object is present in a specific grid cell. This function is also responsible for the bounding box coordinate error. We provide the implementation-specific details of the proposed method in the following section.

## Range and Azimuth parameters

We implement our proposed method using *Driving scenario designer app*. Real-time driving scenarios are generated and data is collected using this app. Later, we implement our technique using the generated radar sensor data in these driving scenarios. Finally, we evaluate our algorithm and present the *precision* and *mean IOU* values in the next section.

### Driving scenario designer app

*Driving scenario designer app* is utilized to test algorithms in real-time driving scenarios. It allows users to create roads and actors using a drag-and-drop menu. It facilitates users to configure sensors like radar, LiDAR, and vision; these sensors help to detect the surrounding objects and collect the information about environment. This application also helps users export sensor data generated during simulation to calculate different parameters necessary for safe driving.

It is worth mentioning that the data generated by the app is trusted by the research community because the app uses a wide range of data sources to generate scenarios, including real-world driving data obtained from various locations and environments. The data collection process follows rigorous protocols to maintain data integrity and reliability. To ensure diversity and coverage, the app incorporates different driving conditions, such as urban, rural, and highway scenarios. This is why the data generated by the app is widely used by the research community^43–45^.

### Data collection

We collect data from the radar sensor mounted on a reference vehicle in real-time traffic scenarios generated using *driving scenario designer app*. Later, we implement our proposed technique on this data and report the precision and IOU value. It is worth mentioning that Driving scenario generator app for creating simulations that reflect real-time traffic scenarios. The app has been used as a robust and dependable platform for the evaluation and optimization of traffic management strategies by the research community. Moreover, the app offers a variety of traffic environments, that include urban, rural, highway, and underway, to name a few. Moreover, the data generated are of real-time nature, and can directly be used to analyze the environment in real-time. It is because of this reason that the app has been widely used by the research community for validating their algorithms^43–45^. The radar sensor details are explained below.

### The radar sensor details

**Figure 13:**
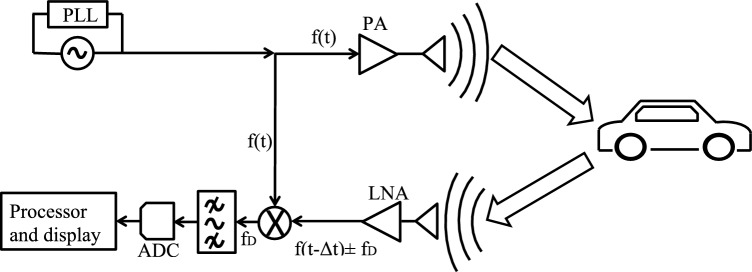
Radar sensor signal processing mechanism.

In our simulation, FMCW radar systems having frequency 76–81 GHz^46^ are used in the automotive industry for object detection and classification. FMCW may contain Multiple Input and Multiple Output (MIMO) antennas system along with many transmission and receiving antennas. Whenever a Radio Frequency (RF) signal is transmitted from a radar sensor, it strikes the surface of objects lying in the Line of Sight (LoS). A portion of the RF signal is reflected to the receiving antennas and merged with transmitted signals. These reflected signals are used to compute the direction, size, angle, and speed of target^46^ as shown in Fig. 13.

To understand the proposed method, from unlimited driving scenarios most commonly faced two traffic scenarios are discussed in Sections “Failure scenario-1 and proposed solution based on proposed strategy” and “Failure scenario-2 and proposed solution based on proposed strategy”.

### Failure scenario-1 and proposed solution based on proposed strategy

**Figure 14:**
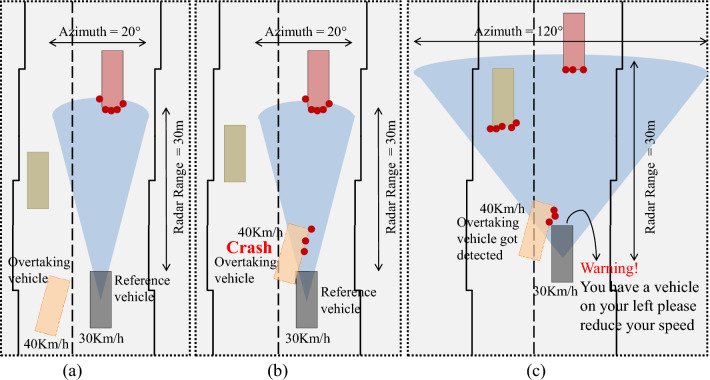
Failure scenario-1 when reference vehicle is moving at 30 Km/h, and proposed solution.

A real-time failure scenario and its solution based on our proposed method have been presented in Fig. 14. In this scenario, a reference vehicle equipped with a radar sensor having the static value of range (30 m) and azimuth (20°) is moving at the speed of 30 Km/h. An overtaking vehicle moving at 40 Km/h speed is trying to change its lane i.e., moving from left to right lane as shown in Fig. 14a. Overtaking vehicle is not in range of the radar sensor mounted on the reference vehicle and therefore the reference vehicle is unaware of overtaking vehicle. Vehicles are moving forward, overtaking vehicle is got detected by the reference vehicle as shown in Fig. 14b but it is too late for the reference vehicle to apply brakes which results in an accident. The reason for this collision is merely the late detection of overtaking vehicle by the reference vehicle.

The solution for the failure scenario-1 based on our proposed method is presented in Fig. 14c. When overtaking vehicle is moving forward and tries to change its lane, the azimuth value of the radar sensor is shifted dynamically i.e., 120°s based on the proposed logic i.e., when the speed of the reference vehicle is up to 40Km/h the azimuth value of radar sensor would be 120°. When overtaking vehicle comes near the reference vehicle due to a dynamic shift of azimuth value, it gets detected and a warning message is generated against detection. As a result, brakes are applied on the reference vehicle and accidents can be avoided.

### Failure scenario-2 and proposed solution based on proposed strategy

**Figure 15:**
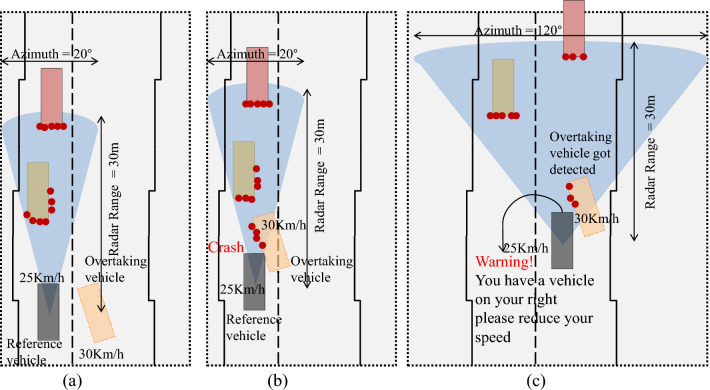
Failure scenario-2 when reference vehicle is moving at 25 Km/h speed, and proposed solution.

Another real-time, lane-changing failure scenario and solution for such scenarios based on our proposed method are presented in Fig. 15. In this scenario, shown in Fig. 15, it can be seen that an overtaking vehicle is trying to overtake the reference vehicle and changing its lane. In this real-time traffic scenario, the reference vehicle is moving at the speed of 25 Km/h while the overtaking vehicle is moving at the speed of 30 Km/h. From Fig. 15a, it can be seen that overtaking vehicle is trying to change its lane and the reference vehicle equipped with a radar sensor having 30 m range, and 20° azimuth value, is not able to detect overtaking vehicle. In the next phase, Fig. 15b it can be seen that overtaking vehicle is got detected by the radar sensor but this detection is too late and an accident takes place. The reason behind this collision is the same i.e., late detection of overtaking vehicle.

Based on the proposed method, the solution to avoid accidents in such a situation is presented in Fig. 15c. The azimuth value of a radar sensor mounted on the reference vehicle is shifted dynamically i.e., when the speed of the reference vehicle is up to 40 Km/h, the azimuth value of the radar sensor will be 120°. As a result of this dynamic shift of azimuth based on the speed of the reference vehicle, overtaking vehicle is got detected in time and a warning message is generated against this detection for the reference vehicle. Due to the timely detection of overtaking vehicles, brakes can be applied and the accident can be avoided.

## Results and discussions

We generate real-time traffic scenarios using *Driving scenario generator app*. A single radar is mounted on a reference vehicle to get the complete traffic details from the right corner, left corner, and the front of the vehicle. From these scenarios, it can be seen that while using static range and azimuth value of radar, there is a high probability of accidents. In particular, when the reference vehicle uses short-range radar or less azimuth value, there may not be enough time for the reference vehicle to apply brakes even if a vehicle is got detected resulting in accidents and causalities. On the other hand, if we use the dynamic range and azimuth value of the radar sensor mounted on the reference vehicle, it can detect surrounding vehicles in time, and thus accidents can be avoided, which can be observed in Figs. 14 and 15. A vehicle moving at a slow speed needs to know only nearby vehicles for safe driving. In such scenarios, a long-range radar may not be needed due to unnecessary computations and wastage of resources that may degrade precision and IOU value.

### Calculation of distance error

The vehicle’s position is calculated using radar. It is worth noting that every reading of a radar contains {($x_i^t,y_i^t,V_i^t,\theta _i^t$), ...} for detection *i*, where *x* is the front distance from the reference vehicle to the target vehicle, *y* is the lateral distance from the reference vehicle to the target vehicle, *V* is the Doppler velocity, and $\theta$ is the vehicle’s heading. The relative position of a target vehicle as seen from the reference vehicle is supplied in the reference vehicle coordinate frame, which is defined at the foremost center point of the vehicle with the x-axis pointing in the driving direction, the y-axis perpendicular to it in the left direction and the z-axis pointing up. The relative position is the three-dimensional vector pointing from the origin of the reference coordinate system to a predefined point in the target vehicle.

**Figure 16:**
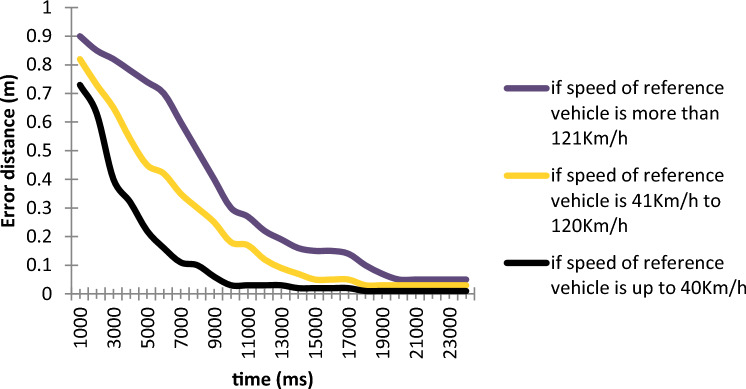
Error distance with different speeds of the reference vehicle.

We plot the error rate in distance calculation against different speed ranges of the reference vehicle in Fig. 16.

It is observed from Fig. 16 that if speed of the reference vehicle is up to 40 Km/h, the rate of distance error is lowest. With the increase in the speed of the reference vehicle, the average rate of distance error increases i.e., if speed of reference vehicle is from 41 to 120 Km/h, the distance error rate is comparatively higher which increase with the increase in the speed of the reference vehicle. When the speed range is highest i.e., 121 Km/h or higher, the average distance error is also highest.

### Calculation of radar sensor average precision

Average precision is a parameter to estimate the performance of an object detection model. Average precision is calculated over recall values on a scale of 0 to 1. *Precision* is the extent to which one can find *true positives (TP)* out of all positive predictions (TP + FP) i.e., including true positives and false positives.

3 $$\begin{aligned} Precision = {(TP)}*{(TP+FP)} \end{aligned}$$

How well one can find *true positives (TP)* out of *all predictions (TP + FN)* is labeled as *recall*.

4 $$\begin{aligned} Recall = {(TP)}*{(TP+FN)} \end{aligned}$$

Weighted mean of precisions at each threshold is labeled as *average precision*. Simply, we can say that *true positives (TP)* out of *total detections (TP + FP)* is the average precision.

5 $$\begin{aligned} Average\_Precision = \frac{TP}{TP+FP} \end{aligned}$$

**Figure 17:**
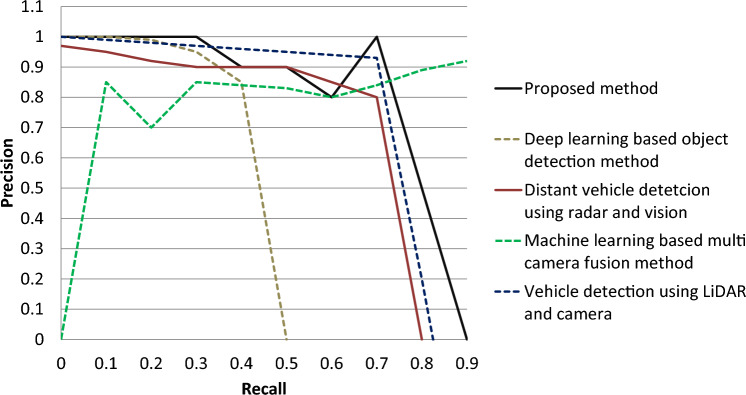
Comparison of average precision.

The authors in previous studies^13,14,20,25,26,29–32^ have calculated the value of average precision for different data sets but they are unable to achieve a reliable value of average precision in real-time settings. On the other hand, the proposed method is able to achieve a higher and reliable value of average precision i.e., 80.0% as shown in Fig. 17. These results are calculated against real-time traffic scenarios and thus the proposed method can be adopted in real-world settings confidently.

**Figure 18:**
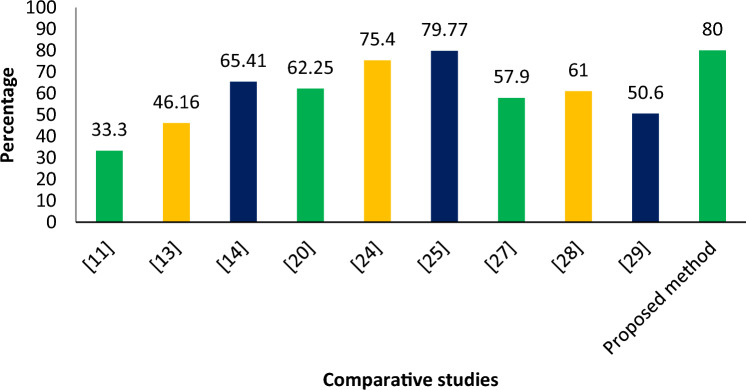
Precision comparison with the previous studies.

The precision value of the proposed method is compared with the state-of-the-art in Fig. 18. It can be seen that the proposed method achieves a higher value of average precision as compared to the previous vehicle position estimation methods.

### Calculation of radar sensor mean IOU

IOU can be defined as an evaluation metric used to estimate the accuracy of an object detection model on a given data set.

**Figure 19:**
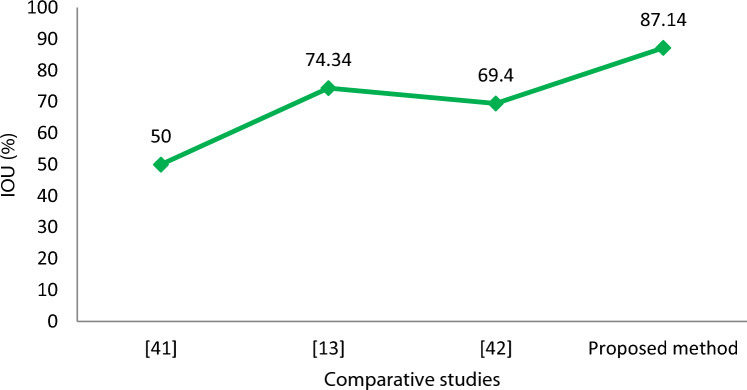
IOU comparison of the proposed method with the previous works.

*IOU* is the ratio of the area of overlap between the predicted bounding box to the ground truth bounding box. The authors in^47^ have also considered IOU for vehicle position estimation and their method offers the IOU value of 50.0% which is not reliable in real-time traffic scenarios. Other works in^13^ and^48^ offer IOU values of 74.34% and 69.4% respectively. Our proposed method based on the dynamic value of range and azimuth values offers better performance in terms of IOU (87.14%) as compared to previous studies presented in Fig. 19.

One of the limitations of the proposed work is that it considers only one-way traffic on highways or motorways and does not study the traffic coming from the opposite direction. Secondly, finding a suitable data set to evaluate the algorithm is challenging as open-source radar sensor data sets are not available. Although data sets are available in a few cases, however, they put forward many restrictions and data synchronization problems.

## Conclusion and future work

In vehicular networks, precise vehicle position estimation is a core factor for safe driving. Previous studies utilize static range and azimuth value of a radar sensor for position estimation. However, these methods face problems in precise vehicle position estimation particularity in harsh weather conditions. In this paper, we propose a vehicle position estimation method based on dynamic range and azimuth value of a radar sensor. Our results show that proposed method improves the vehicle position estimation results significantly. Proposed method is utilizing dynamic range and azimuth values of radar sensor rather than static parameters. This study concludes that traffic accidents can be reduced significantly based on the proposed method. Radar precision (80.0%) and mean IOU (87.14%) calculation based on our proposed strategy are much better as compared to the previous algorithms using multiple sensors. Data fusion complexity by using multiple sensors is also avoided by using proposed method.

In the future, this work can be extended to urban and two-way traffic scenarios and can be implemented in real-world traffic scenarios. The proposed work can also be enhanced to incorporate harsh weather conditions i.e., lightening, fog, and snowy weather. Additionally, in the future, proposed method can be developed to consider the road hurdles, pot holes, objects and surrounding animals. Finally, security aspects of the vehicles can be considered^49^.

## Data Availability

The datasets used and/or analysed during the current study are available from the corresponding author on reasonable request.
